# Relationship between global identity and pro-environmental behavior and environmental concern: a systematic review

**DOI:** 10.3389/fpsyg.2023.1033564

**Published:** 2023-04-17

**Authors:** Vivien Pong, Kim-Pong Tam

**Affiliations:** Division of Social Science, The Hong Kong University of Science and Technology, Hong Kong, Hong Kong SAR, China

**Keywords:** global identity, identification with all humanity, global citizenship, world citizen, global belonging, pro-environmental behavior, environmental concern, systematic review

## Abstract

Global issues such as environmental problems and climate change, require collective efforts. Global identity has been linked to the promotion of pro-environmental behavior by international and environmental organizations. In environment-related research, this all-inclusive social identity has been consistently related to pro-environmental behavior and environmental concern, but the underlying mechanisms are not well understood. This current systematic review seeks to examine past studies across disciplines that have reported findings on the relationship between global identity and the constructs of pro-environmental behavior and environmental concern and to synthesize findings on the potential pathways behind this relationship. Thirty articles were identified through a systematic search. We found that most studies reported a positive correlation, and the effect of global identity on pro-environmental behavior and environmental concern was stable across studies. Only nine of the studies empirically examined the underlying mechanisms of this relationship. Three major themes of these underlying mechanisms emerged: obligation, responsibility, and relevance. These mediators highlight the role of global identity in pro-environmental behavior and environmental concern *via* how individuals relate to other humans and how they appraise environmental problems. We also observed a heterogeneity in measurements of global identity and environment-related outcomes. As a topic of interest in multiple disciplines, a variety of global identity labels have been adopted, such as global identity, global social identity, humanity identity, Identification With All Humanity, global/world citizen, connectedness to humanity, global belonging, and psychological sense of global community. Self-report measures of behavior were common, but observations of actual behavior were rare. Knowledge gaps are identified, and future directions are suggested.

## 1. Introduction

Environmental problems do not recognize borders; their impacts reach all of us across countries and generations, regardless of race or other individual characteristics. Tackling environmental problems and climate change requires world-wide efforts. Organizations appeal to the public to take collective action to combat global problems by reminding us that we are members of the world. Organizations such as Pure Earth and One Community used slogans such as “Pollution knows no borders” (Bernhardt et al., 2019) and “One Planet. One Home. One Community” (One Community, n.d.) to raise awareness and call for action. International organizations such as UNESCO and the United Nations have also promoted the role of global citizenship in education to facilitate international cooperation and promote social transformation (UNESCO, 2014) and achieve the Sustainable Development Goals (United Nations, 2015).

In environment-related research, the concept of global identity has been gaining increased attention over the past decade (also see review by McFarland et al., 2019). Global identity refers to an all-encompassing, all-inclusive form of group identity, wherein all humans, regardless of race, religion, sexual orientation, and other identifiers, are seen as one group. It has been studied across scholarly disciplines and found to be positively related to pro-environmental behavior (PEB) and environmental concern (EC). However, the mechanisms underlying this relationship are not well understood. The present systematic review aims to synthesize existing findings that have examined the relationship between global identity and PEB and EC and to identify directions for future development in this area of study.

### 1.1. The construct of global identity

The social identity perspective suggests that group membership guides behavior when membership is salient (Tajfel and Turner, 1979; Turner et al., 1987). In general, individuals are concerned about people who they consider ingroup members and adjust their behavior to serve the ingroup’s interests and the welfare of other members (Reese, 2016). In addition, based on the cost-reward model, one would have a great feeling of responsibility for the welfare of another person when the person is perceived to be similar to the self (Dovidio et al., 1991), and ingroup members are perceived as more similar to the self than outgroup members. Empirical evidence indicates that when a more inclusive level of social group membership is made salient (e.g., a fan of football rather than a fan of a specific football team), people even consider formerly rival outgroup members (e.g., a rival football team) as ingroup members and would be as likely to offer help (Levine et al., 2002, 2005).

According to the social identity theory and self-categorization theory, individuals categorize themselves as members of a higher-order social unit and identify themselves with groups on different levels of inclusiveness (Tajfel and Turner, 1979; Turner et al., 1987). There are three levels of inclusiveness in self-categorization (Turner, 1982): The base level is the interpersonal level of differentiating oneself from another ingroup member; the intermediate level is based upon differentiating the ingroup members from outgroup members; and the highest level is the categorization of oneself as part of all humanity. Global identity reflects the highest, all-inclusive level of self-categorization.

Until recently, global identity has received little empirical attention (Reysen, 2022). In two reviews (McFarland et al., 2019; Reysen, 2022) and one empirical analysis (McFarland and Hornsby, 2015), 12 measures of global identity were identified and about half of which were published after the year 2010. Overall, as a construct, global identity was negatively associated with ethnocentrism, authoritarianism, social dominance orientation, and self-centeredness, and positively associated with dispositional empathy, openness to experience, and values of universalism, care, and justice (Hamer et al., 2019). Over the past decade, there has been a surge in interest in global identity and overall, global identity was found to be related prosociality. For example, global identity predicted humanitarian concerns (McFarland and Hornsby, 2015) and intergroup forgiveness (Hamer et al., 2018), was related to willingness to provide humanitarian help to people in COVID-affected countries (Deng, 2021) and those who suffered from natural disasters (Sparkman and Hamer, 2020), and was associated with greater engagement with the global community, given that globalization was perceived as positive (Reysen, 2022).

### 1.2. The role of global identity in PEB and EC

As discussed above, the social identity perspective suggests that individuals tend to care about and act in the interest of ingroup members’ welfare. Indeed, past findings support the link between social identity and PEB. For example, identification with a pro-environmental initiative that promoted sustainable, low-carbon living that led to local energy autonomy predicted participation intention in the initiative (Bamberg et al., 2015). Similarly, if individuals perceived ingroup members as negatively impacted by environmental problems, they are more likely to take action to mitigate the problems when they strongly identify with the group. For example, identification with the local community was found to be positively related to the intention to participate in a neighborhood initiative for climate protection (Rees and Bamberg, 2014).

In the past decade, there has been a surge of studies on the relationship between global identity and PEB and EC. Global identity has been linked to the promotion of PEB and the mitigation of environmental problems and climate change. Studies were found not just in psychology (Reysen, 2022) but also in business (Russell and Russell, 2010), political science (Chung and Milkoreit, 2021), education (Wynveen et al., 2012), and tourism (Proyrungroj, 2022). Given the growing interests in global identity and PEB and EC, an in-depth systematic review of our understanding of this relationship thus far is warranted. However, to the best of our knowledge, such a systematic review has yet to be done. A review by Reysen (2022) was informative; however, it was not a systematic review and therefore its goal was not to systematically examine the relationship between global identity and the constructs of PEB and EC. Although Reysen (2022) referred to several studies that examined PEB and its related constructs (Reysen and Katzarska-Miller, 2013; Der-Karabetian et al., 2014, 2018; Der-Karabetian and Alfaro, 2015; Rosenmann et al., 2016), they were discussed in relation to the argument regarding globalization. The present systematic review seeks to fill this gap in the literature by systematically examining studies to understand the relationship between global identity and the constructs of PEB and EC. The goal of the current systematic review is twofold: (1) to examine the empirical evidence to date on the relationship between global identity and environmental outcomes/variables and (2) to examine the underlying mechanisms of this relationship. The current review also identifies issues related to measurements and methodology and suggests future directions.

## 2. Methods

### 2.1. Databases

Since there is a lack of guidelines as to how databases should be chosen when conducting a systematic review, we chose them based on two principles: one is to choose databases that are conventionally used and second is to consider the topic at hand in relation to the relevant disciplines involved. As such, we referred to other systematic reviews in the field of psychology and expanded the disciplines to also include sociology, education, political science and social sciences in general. The shortlisted databases identified included APA PsycINFO, APA PsycARTICLES, Academic Search Premier, ERIC, and GreenFILE under EBSCOhost, as well as Scopus, ProQuest and Web of Science.

### 2.2. Search terms

Two sets of search terms were used: one related to global identity and the other related to PEB and EC. For global identity, we combed through the keywords and the content of reviews such as the one that empirically compared different global identities by McFarland and Hornsby (2015) and other scale development studies such as the local-global identity measures (Tu et al., 2012). For PEB and EC, we referred to the search terms used in other relevant systematic reviews (e.g., Udall et al., 2020).

Examples of global identity search terms are: “all humans everywhere,” “global citizen*,” “Global Belonging” and so on. Examples of environmental-related construct search terms include general behavior (e.g., “green consumer behav*,” “pro-environmental behav*”), behavior of specific domain (e.g., “waste recycling behav*,” “energy conservation,” “travel mode choice”), environmental issues (e.g., “climate change” and “global warming”), attitude (e.g., “environmental attitude*,” concern (“environmental concern”), and values (e.g., “ecologic value*”). All search terms are in quotations to search the specific terms. Wildcards were used to cover different forms of the same term. For a full list of the search term, please see Section 1 “Search terms” of the Supplementary material).

Since dozens of search terms were involved for each construct and varying string lengths in different databases were a concern (Gusenbauer and Haddaway, 2020), a test on the search term string length was performed before any search was conducted in each database. The result of this test showed that the string length limit was not reached for any of the databases and all the search terms were included in each search. Details of this test are reported in Section 2 “Search term string length test” the Supplementary material.

### 2.3. Search strategies

All searches consisted of both sets of search terms entered with the same field (for example “abstract,” “title” or “Keywords”). When possible, we expanded the search to apply equivalent subjects (APA PsycINFO, APA PsycARTICLES, Academic Search Premier, ERIC, and GreenFILE in EBSCOhost). Limiters were set to scholarly (peer reviewed) journal (exclude book reviews), English, journal article (exclude dissertation), human/male/female (exclude animal). Similar expanders and limiters were used in all other databases (Scopus, ProQuest and Web of Science). The detailed settings and the number of articles returned corresponded to each database can be found in Section 3 “Search settings and related details” in the Supplementary material. A total of 1,069 articles were yielded from all eight databases. The search was completed on the same day–April 13, 2022. As such, all articles that were published on the day or before were included.

### 2.4. Screening and detailed assessment process

**FIGURE 1:**
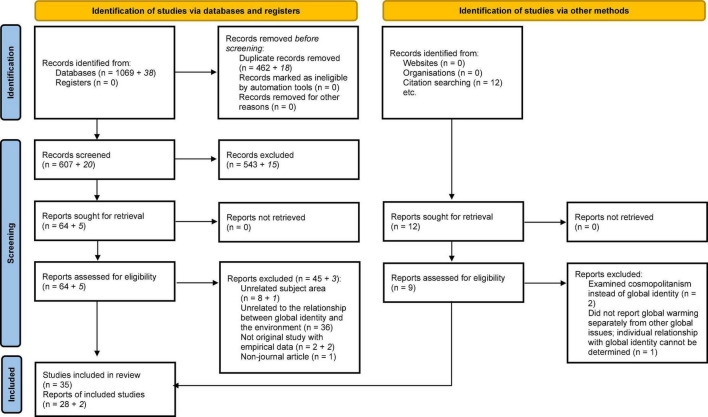
PRISMA 2020 flow diagram for new systematic reviews which included searches of databases, registers and other sources. The numbers in italic (after the “+” sign) represent the articles from the search with the additional keywords. Please refer to section “2.6. Screening process and final inclusion” regarding the screening process and the search details in section “4. Additional search with new search terms” in the Supplementary material. From Page et al. (2021) for more information, visit: http://www.prisma-statement.org/.

The reporting of this systematic review was guided by the standards of the Preferred Reporting Items for Systematic Review and Meta-Analysis (PRISMA) Statement. We did the initial screening of all 1,069 articles, of which 462 duplicates were identified. The first author read all the abstracts of the remaining 607 articles to identify relevant articles. The following inclusion and exclusion criteria were used throughout the screening process. Figure 1 shows the screening process and details in a PRISMA flow chart.

### 2.5. Inclusion and exclusion criteria

Only peer-reviewed journal articles that reported original results with empirical data in English were included. Other types of writing were excluded, such as dissertation (PsycINFO), book review (PsycArticles), book chapter, conference proceedings, unpublished manuscripts, reviews, editorials and commentary. Subject areas that were obviously not related to the current topics were excluded, such as agricultural and biological sciences, engineering, computer science, biochemistry, genetics and molecular biology chemical engineering and so on.

### 2.6. Screening process and final inclusion

After this initial screening process using abstracts, 64 were deemed relevant for further assessment. Upon review of the full-text, 45 of them were excluded based on the above exclusion criteria. The remaining 19 articles were deemed relevant.

Twelve additional articles were found outside of the systematic search. Ten of these additional articles were identified in the process of reviewing the 64 articles—they were found in the reference lists. One additional article was identified in a recent review on global identity by Reysen (2022) and one was a publication by our research team (Chan et al., 2020). Of the 12 additional articles, three articles were excluded: Two of the articles (Grinstein and Riefler, 2015; Leung et al., 2015) examined cosmopolitanism instead of global identity, and one article (Buchan et al., 2011) measured global warming as part of a set of global issues and reported global issues as a total score but did not report separately the relationship between global identity and global warming (or any of the individual global issues).

During our response to a reviewer’s comments, we became aware of two additional keywords related to global identity: “global identification” and “global-level identification.” We then used the terms to look for potential additional sources. To match our main search, we replicated the search in the same databases with the exact same settings and the same PEB and EC search terms, and we limited our search to publications from April 2022 or before. The only difference was that only the two new terms were used for global identity. This second search yielded 38 articles. Using the same inclusion and exclusion criteria and the same screening process, five were deemed relevant for further assessment. Upon review of the full-text, three of the five articles were excluded based on the exclusion criteria and two relevant articles were identified (Furlong and Vignoles, 2021; Aydin et al., 2022). This search result is presented in Section 4 “Additional search with new search terms” in the Supplementary material, separate from the main search results. The results of this additional search are shown in italic as an addition to the main search in the PRISMA diagram (Figure 1).

Based on the above efforts, 30 articles in total were included in this review. Among the 30 articles, 41 studies were reported but six studies were excluded based on the above inclusion and exclusion criteria stated above. The removal of the six studies left 35 studies from the 30 articles in this review.

The first author coded each article to systematically document the global identity label and definition used by the researchers and the measures used for global identity and PEB and EC. The relevant theories, sample characteristics, the major findings, and other details such as publication year were also coded. The findings were synthesized and reported below.

## 3. Results and discussion

In the following subsections, we summarize the findings from the literature regarding the relationship between global identity and PEB and EC. We first reported the overall trend of this relationship. Next, we examined the underlying mechanisms of this relationship by reviewing the theoretical foundations used to explain the relationship and reviewing the evidence from nine studies that empirically tested the explanations of the relationship. Lastly, we included observations that are additional to our main objective but nonetheless relevant and important regarding measurement and sampling issues.

### 3.1. Evidence on the positive relationship between global identity and PEB and EC

We first examined evidence on the relationship between global identity and PEB and EC.

Of the 35 studies reviewed, 30 examined the zero-order correlation between global identity and PEB and EC. Only one study reported no correlation at all (Furlong and Vignoles, 2021); all other studies reported a significant correlation. The results are summarized in Table 1, along with other study information. Five studies did not report any zero-order correlation; the reason was either that global identity (Chung and Milkoreit, 2021) or the construct of PEB/EC was dichotomized (Running, 2013), or that testing the correlation was not the main interest of the study (Russell and Russell, 2010; Strizhakova and Coulter, 2013; Strizhakova et al., 2021). These studies conducted other analyses, such as ANOVA (Chung and Milkoreit, 2021) and logistic regression (Running, 2013), and reported a significant relationship between the two constructs. Most of the 35 studies conducted further analyses, such as regression, with a variety of variables controlled for, and still revealed a significant relationship between global identity and PEB and EC.

**TABLE 1:** Details of 35 studies in 30 articles on global identity and environment-related constructs.

References	Discipline	Sample origin (size) composition	Mean age	Global identity scale	Pro-environmental outcomes	Results
Assis et al., 2017	Environment	United States (*n* = 201) student	*M* = 22.8	Global Citizen [five items; adapted from scales by Doosje et al. (1995) and Reysen et al. (2013)]	Motivation for environmental behavior (23 items; Pelletier et al., 1998)	+ (4 subscales) N.S. (2 subscales)
					Human interdependence with nature (16 items; Corral-Verdugo et al., 2008)	+
					Environmental attitudes (30 items; Pettus and Giles, 1987)	+
					New Environmental Paradigm (12 items; Dunlap and Van Liere, 1978)	+
Aydin et al., 2022	Psychology	**Study 1:** Türkiye (*n* = 1121) **Study 2:** WVS (*n* = 40,330 from 43 countries) community samples	*M* = 35.79	**Study 1:** global identification (two items; adapted from Verkuyten and Yildiz, 2007) **Study 2:** World citizen (one item; WVS, Wave 5, 2005–2009)	**Study 1:** Pro-environmental beliefs (15 items; New Ecological Paradigm; Dunlap et al., 2000) (three items; Climate change beliefs; Stanley et al., 2017). Pro-environmental behavior (items # not reported; Liu and Sibley, 2012; adapted from Lange and Dewitte, 2019). Pro-environmental activism (seven items; adapted from Lange and Dewitte, 2019). **Study 2:** Pro-environmental beliefs (one item; WVS, Waves 5 and 6) Pro-environmental activism (two items; WVS, Waves 5 and 6). Pro-environmental behavior (one item; WVS, Waves 5 and 6)	+
Barth et al., 2015	Psychology	**Study 1:** Germany (*n* = 450) student sample (**Study 2:** excluded)	*M* = 28.86	Global identity (four items; adapted from Leach et al., 2008)	Willingness to engage in collective action on behalf of the victims of climate change injustice (eight items)	+
					Donation for an NGO that fights for more climate change justice by passing on the chance to win a gift coupon (one item)	
Chan et al., 2020	Psychology	Hong Kong (university students; *n* = 62 and university staff members; *n* = 177) mixed sample	Students: *M* = 20.69 Staff: *M* = 32.83	Humanity identity (five items; Buchan et al., 2011; McFarland et al., 2013; Reysen and Katzarska-Miller, 2013; Reese and Kohlmann, 2015)	Intention of turning off lights during Earth Hour (four items; adapted from Fishbein and Ajzen, 2010)	Correlation: + Regression: N.S. Mediation analysis: +
					Actual behavior of turning off lights during Earth Hour (one item)	Correlation: + Regression: N.S.
Chung and Milkoreit, 2021	Political science	United States (students; *n* = 298) WVS (community; *n* = 1249) mixed sample	United States students: *M* = 22.73 WVS: *M* = 45.90	World Citizen (one item; WVS, Wave 5, 2005–2009; student sample: Y/N/IDK and WVS sample: one-item agreement with statement “I see myself as a world citizen” on a four-point scale)	Climate change beliefs (six items)	+
					Perceived severity of climate change (one item; WVS, Wave 5, 2005–2009; student sample: two items)	Student sample: + WVS: N.S.
Der-Karabetian and Alfaro, 2015	Social science	United States (*n* = 117) Netherlands (*n* = 45) Brazil (*n* = 116) Student sample	United States: *M* = 21.1 Netherlands: *M* = 24.6 Brazil: *M* = 32.0	Global belonging (seven items; Der-Karabetian and Ruiz, 1997)	Sustainable behavior (six items on conservation, consumption and recycling; Der-Karabetian et al., 2014)	Correlations: + Regression: United States: N.S. Netherlands: + Brazil: N.S.
Der-Karabetian et al., 2014	Psychology	United States (*n* = 442) Mainland China (*N* = 516) Taiwan (*N* = 164) mixed sample	United States: *M* = 21.9 Mainland China: *M* = 23.8 Taiwan: *M* = 23.2	Global belonging (Der-Karabetian and Ruiz, 1997)	Sustainable behavior (six items on conservation, consumption and recycling)	Correlations: + Regression: United States: + Mainland China: N.S. Taiwan: +
Der-Karabetian et al., 2018	Psychology	United States (*n* = 298) student sample	*M* = 22.73	Global belonging (Der-Karabetian and Ruiz, 1997)	Sustainable behavior (six items; Der-Karabetian et al., 2014)	+
Furlong and Vignoles, 2021	Psychology	United Kingdom (*n* = 203) community sample	*M* = 46.77	Global identification (12 items; adapted from Koc, 2018)	Collective action past behavior (10 items)	N.S.
					Collective action future intentions (10 items)	N.S.
Iwata, 1996	Psychology	Japan (*n* = 156) student sample	Age not found/reported	Scale #1: belief that national borders are needless (four item)	Pro-environmentalism (27 items; Iwata, 1990)	N.S.
				Scale #2: global communities are bounded common fate (five items)		+
Janmaimool and Khajohnmanee, 2020	Environment	Thailand (*n* = 423) student sample	*M* = 20.92	Global citizenship (10 items; Janmaimool and Khajohnmanee, 2020)	Pro-environmental behavior (nine items)	+
Joanes, 2019	Business	United States Germany Sweden Poland (*n* = 4591; breakdown by country not available) mixed sample	*M* = 42.17	IWAH (seven items; McFarland et al., 2012; Reese et al., 2015)	Intention to reduce clothing consumption (two items)	+
Kim et al., 2021	Environment	Korea (*n* = 387) community sample	*M* = 43.84	Global identity [three items; adopted from various measures, including Tu et al. (2012) on local-global identity]	Waste reduction intention (two items) Water saving intention (two items)	+
Lee et al., 2015	Psychology	Canada (*n* = 324) student sample	*M* = 19.7	IWAH (nine items; McFarland et al., 2012)	New Ecological Paradigm (NEP; 15 items; Dunlap et al., 2000)	Significance level not reported (*r* = 0.14)
					Pro-environmental Behavior (18 items; Kaiser et al., 2007)	+
Leithead and Humble, 2020	Education	Ghana (*n* = 141 from 3 schools) Student (children) sample	*M* = 11.0	Global citizenship identification (two items; Leithead and Humble, 2020)	Belief in environmental sustainability (two items; Reysen et al., 2012)	+
Loy and Reese, 2019	Psychology	Germany (*n* = 258) Mixed sample	*M* = 27.0	IWAH–adapted version (10 items; McFarland et al., 2012; Reese et al., 2015)	General Ecological behavior (24 items; Kaiser and Wilson, 2000, 2004); Climate policy support (adapted from European Social Survey, Tobler et al., 2012)	+
Loy et al., 2021a	Psychology	**Study 1:** Germany (*n* = 498) **Study 2:** United Kingdom (*n* = 508) community samples	**Study 1:** *M* = 48.1 **Study 2:** *M* = 47.5	**Study 1:** IWAH (nine items; McFarland et al., 2012; Reese et al., 2015) **Study 2:** Situational IWAH (10 items; McFarland et al., 2012)	**Study 1:** General Ecological behavior (25 items; Kaiser and Wilson, 2000, 2004) **Study 2:** Information viewing and duration; Support for climate initiatives and budget allocated; General Ecological behavior (24 items; Kaiser and Wilson, 2000, 2004)	+
Loy et al., 2021b	Psychology	Students and social media groups in Germany (*n* = 317) (range = 18 to 65) community sample	*M* = 28.4	IWAH–adapted version (10 items; McFarland et al., 2012; Reese et al., 2015; Loy and Reese, 2019)	Flight-related CO2 emissions (one item)	Self-definition: N.S. Self-investment: -
					Refraining from flying (one item)	N.S.
					Flight shame (two items)	+
					Willingness to pay for carbon offsetting (one item)	+
					Amount of compensation on flight-related CO2 (one item)	+
					Support for decarbonized mobility policies (11 items; Loy and Reese, 2019)	+
Loy et al., 2022	Psychology	Germany (*n* = 401) 42.5 (range = 18 to 82) community sample	*M* = 42.5	IWAH (10 items; adapted from McFarland et al., 2012)	General Ecological Behavior Scale (34 items; adapted from Kaiser and Wilson, 2000, 2004). Climate Policy support (10 items; Loy and Reese, 2019)	+
Ng and Basu, 2019	Psychology	**Study 1:** WVS (*n* = 75,934 from 56 countries) **Study 2:** Singapore (undergraduate students; *n* = 226) **Study 3:** Singapore (UG students; *n* = 96) **Study 1:** community sample **Study 2** and **3:** Student samples	**Study 1:** *M* = not found/reported **Study 2:** *M* = 21.68 **Study 3:** *M* = 22.07	**Study 1:** World citizen (one item; WVS, Wave 5, 2005–2009) **Study 2:** local-global identity (four items; Tu et al., 2012) **Study 3:** manipulation of global identity	**Study 1:** Personal responsibility toward the environment (one item; WVS, Wave 5) **Study 2:** Willingness to pay for printed materials to be printed on recycled paper (one item) **Study 3:** Willingness to pay for printed materials to be printed on recycled paper (one item)	+
Pong, 2021	Psychology	United States (*n* = 465) community sample	*M* = 38.23	IWAH (McFarland et al., 2012)	Food waste reduction intention (eight items)	+
					Policy support (four items)	+
					Behavioral proxies (two items)	N.S./+
Renger and Reese, 2017	Psychology	Germany (*n* = 469) community sample	*M* = 30.8	Global identity [four items; adapted from Reese and Kohlmann (2015), Walker et al. (2015)]	Behavioral intentions (three items; Fielding et al., 2008). Self-report current donation to pro-environmental organization (one item)	+
Reysen and Hackett, 2016	Psychology	**Study 1:** United States (*n* = 239) student sample (**Studies 2** and **3:** excluded)	*M* = 25.39	IWAH (McFarland et al., 2012; Reese et al., 2015)	Environmental sustainability as prosocial values (two items, **Study 1**; Reysen et al., 2012)	+
Reysen and Katzarska-Miller, 2013	Psychology	**Study 1:** United States (*n* = 726) **Study 2:** U.S. (*n* = 1202) student sample	**Study 1:** *M* = 28.9 **Study 2:** *M* = 25.86	Global citizenship identification (two items; adapted from Reysen et al., 2012)	Environmental sustainability (two items)	+
Running, 2013	Social Science	WVS (*n* = 40,330 from 43 countries) community sample	*M* = 41.84	World Citizen (one item; WVS, Wave 5, 2005–2009)	Concern for global warming (one item; WVS, Wave 5, 2005–2008)	+
Russell and Russell, 2010	Business	**Study 1:** United States (*n* = 75) student sample (**Studies 2** and **3:** excluded)	*M* = not found/reported	Global identity (six items; Russell and Russell, 2010)	Purchase intention of a sustainable brand among 13 other listed brands (one item; **Study 1**)	+
Scafuto, 2021	Psychology	United States (*n* = 277) student sample	*M* = 19.36	Psychology sense of global community (four items; Hackett et al., 2015)	Climate Change response efficacy (Li and Monroe, 2018; Scafuto et al., 2018)	+
Strizhakova and Coulter, 2013	Business	Brazil = 319 Russia = 328 India = 305 China = 295 United States = 302 Australia = 323 Australia = 40 *equal number of participants in each of three age groups: 18–30, 31–45, and 46–60; community samples	Brazil: *M* = 37 Russia: *M* = 37 India: *M* = 38 China: *M* = 34 United States: *M* = 39	Global connectedness (seven items; a subscale—one of three dimensions of global cultural identity; Strizhakova and Coulter, 2013)	Concern for environmentally friendly products (three items*) Willingness to pay extra for environmentally products (three items*) Perceptions of global companies as environmentally friendly (six items*) Likelihood to engage in PEB (four items)	Emerging market: + Developed market: N.S.
Strizhakova et al., 2021	Business	**Study 2:** United States MTurk (*n* = 450) community sample (**Study 1** was excluded)	**Study 2:** *M* = 34	Global identity (four items; adapted from Strizhakova and Coulter, 2013)	Purchase intention of a fictitious environmentally friendly brand (two items; **Study 2**)	+
Woosnam et al., 2019	Environment	United States college students (*n* = 426) United States community (*n* = 220) mixed sample	*M* = 24.5	Global citizenship identification (22 items; Reysen et al., 2012)	Future environmental volunteering intentions (eight item; adapted from Zaichkowsky, 1985; Sparks et al., 1997)	+

WVS, World Value Survey. For the measurements of global identity and pro-environmental outcomes, if the scales were developed by the authors in the study, no citation is included. In the “Results” column, “+” signs indicate positive and significant results from any analysis (e.g., correlation, regression) regarding the relationship between global identity and the corresponding environment-related outcomes. A “-” sign indicates a negative relationship and “N.S.” signifies non-significant results.

*The items were adapted based on Cornelissen et al. (2008); Webb et al. (2008); Kilbourne et al. (2009).

Among these 35 studies, a wide range of PEB and EC measures were used. The most common type was behavior-related measures, such as intentions to engage in PEB, self-report private-sphere behavioral habits, self-report past behavior, policy support, willingness to pay, and participation in protests. Some studies also included other environment-related measures such as pro-environmental values (Reysen and Hackett, 2016), climate change beliefs (e.g., Chung and Milkoreit, 2021), environmental attitude (e.g., Assis et al., 2017), EC (e.g., Running, 2013), and perceptions (e.g., perception of severity of climate change and perception of whether a global brand was environmentally friendly; Strizhakova and Coulter, 2013).

Overall, it can be concluded from the existing research that global identity is robustly positively associated with stronger engagement in environmental issues. However, further analyses revealed two nuances to consider. We now discuss these nuances individually and offer recommendations for future research.

1. Cross-national differences in this relationship were documented, even in studies with the same research design and the same set of constructs. Across six samples in two studies, Der-Karabetian and Alfaro (2015) examined the relationship between global belonging and PEB, together with five other constructs: globalization general impact, globalization impact on own country, national belonging, world-mindedness, and personal environmental risk (Der-Karabetian et al., 2014; Der-Karabetian and Alfaro, 2015). A significant positive relationship between global identity and PEB in zero-order correlation was found in all samples. But when the five constructs were controlled for in a regression analysis, global identity significantly predicted PEB in only half the samples: in one of the US samples, Taiwan, and Netherlands but not in mainland China (Der-Karabetian et al., 2014), and not in the other US sample or Brazil (Der-Karabetian and Alfaro, 2015). These findings from two studies with the same design suggest that the relationship between global identity and PEB and EC may vary in different societal or cultural contexts when the same set of constructs were controlled for. Based on this observation, *we recommend that researchers use a diverse sample of countries to more systematically document and explain potential cross-national or cross-cultural variability of the relationship* [*Recommendation #1*].
2. Two studies reported a significant indirect relationship between global identity and PEB, even when the direct effect of global identity on PEB was not significant (Chan et al., 2020; Furlong and Vignoles, 2021). In Chan et al. (2020), global identity only indirectly predicted the intention to turn off non-essential lights and the actual light-off behavior during the Earth Hour event (Chan et al., 2020). The researchers suggested that, when predicting a highly specific behavior (i.e., turning off non-essential lights during an event), domain-general motivational factors (e.g., global identity) might exert their effects on behavior indirectly through the influence on domain-specific motivational factors (e.g., behavioral attitude). Furlong and Vignoles (2021) observed that global identity was indirectly associated with participation in an environmental movement *via* moral conviction (defined as a strong and absolute stance on moral issues), anger, and movement-specific identity. *We recommend researchers explore the indirect effect of global identity by considering the underlying mechanisms of the relationship between global identity and PEB/EC [Recommendation #2]* (see also section “3.3. Evidence on the mediating mechanisms underlying the positive relationship”).

### 3.2. Theoretical explanations of the observed positive relationship

Sixteen of the 30 articles explicitly referred to the social identity theory to explain the role of global identity in PEB and EC. This theory posits that, when individuals define themselves as a member of a social group, they are more likely to care about the welfare of members of the group and act in the interest of the group. As such, there are emotional (such as concern for the group) and behavioral (such as prosocial and cooperative behavior) implications of social identity. Six other articles implicitly referred to the social identity theory: Although the social identity theory was not explicitly mentioned, the definitions of global identity based on the social identity theory were cited, and the studies considered the same attributes and implications of the theory (e.g., Leithead and Humble, 2020). In total, we observed that 22 articles referred to the social identity theory when explaining why global identity was expected to be associated with PEB and EC.

It is noteworthy that five of the 22 articles integrated the social identity theory with other theories or frameworks, including the norm activation theory (Joanes, 2019; Kim et al., 2021), the theory of planned behavior (Chan et al., 2020), the social identity model of collective action (Barth et al., 2015; Furlong and Vignoles, 2021), and the encapsulated model of social identity in collective action (Furlong and Vignoles, 2021). All of these five studies conceptualized the role of global identity as an antecedent of the more proximal predictors of behavior. For instance, Joanes (2019) and Kim et al. (2021) conceptualized global identity as the antecedent of ascribed responsibility and personal norm, two key concepts in the norm activation theory, when understanding reduced clothing consumption (Joanes, 2019) and waste reduction and water saving intentions (Kim et al., 2021). Similarly, Chan et al. (2020) referred to the theory of planned behavior and considered moral norm and attitude the pathways through which global identity predicted participation in Earth Hour.

The remaining studies referred to other relevant theories and concepts. These can be grouped into three perspectives: interconnectedness, common threat, and global focus. For the interconnectedness perspective, the theories share the idea that the world is a single community, bound together by a common fate. As members of the community, we have a responsibility to the community and other members. These theories and concepts include the triangular model of responsibility (Ng and Basu, 2019), bound by common fate (Iwata, 1996), and sense of community (Woosnam et al., 2019; Scafuto, 2021). For common threat, Der-Karabetian et al. (2014, 2018) and Der-Karabetian and Alfaro (2015) referred to the superordinate goal theory, which suggests that the desire to eliminate perceived common threats as a group leads to cooperation, solidarity, prosociality and group identity reinforcement. Lastly, for global focus, Strizhakova and Coulter (2013) and Strizhakova et al. (2021) referred to the global consumer cultural identity theory, which suggests that individuals with a strong cultural identity are more aware of global (vs. local) consumer culture, which includes concern about the welfare of the global environment. Similar to the social identity theory, these three perspectives explicate the behavioral motivations of global identity.

### 3.3. Evidence on the mediating mechanisms underlying the positive relationship

We next review the nine studies that empirically examined the mediating psychological mechanisms underlying the positive relationship between global identity and PEB and EC. All these studies considered at least one mediator (Table 2). Based on our coding, we identified three main categories of mediators: obligation (*n* = 4), responsibility (*n* = 4), and relevance (*n* = 2). Other mediators appeared in one study only: attitude (Chan et al., 2020), awareness of needs and outcome efficacy (Joanes, 2019), and moral convictions, anger, and environmental movement-specific identity (Furlong and Vignoles, 2021).

**TABLE 2:** Summary of the nine studies that empirically examined the relationship between global identity and environmental-related construct.

References	Mediator(s) (scale)	Mediator coded	Theories/Models and explanation
Barth et al., 2015	Solidarity (four items; Likki and Staerklé, 2014)—a form of collective responsibility that motivates humans to take care of the more vulnerable members of a community	Responsibility	Model: SIMCA Explanation: The encompassing quality of global identity generates responsibility toward disadvantaged humans regardless of their nationality and other characteristics. Group membership leads to collective action with the aim to improve the group’s conditions *via* solidarity along with other factors based on SIMCA.
Chan et al., 2020	Moral Norm (three items; adapted from Kaiser and Scheuthle, 2003). Attitude (six items; written based on guidelines of TPB; Fishbein and Ajzen, 2010)	Moral obligation attitude	Theories: SIT + TPB Explanation: Humanity identity guides prosocial behavior based on SIT. As a general-domain construct, humanity identity is found to associate with positive evaluation of environmental sustainability and moral obligation toward performing a behavior, which in turn, predict domain-specific behavioral intentions based on TPB.
Furlong and Vignoles, 2021	Moral Conviction (four items; van Zomeren et al., 2019). Anger (two items; adapted by Rees and Bamberg, 2014; van Zomeren et al., 2019). Environmental Movement-Specific Identity (12 items; Koc, 2018)	Moral convictions anger environmental movement-specific identity	Model: SIMCA + EMSICA Explanation: Global identity implies greater sensitivity to injustice of climate change and identification with the victims of climate change who may be geographically and temporally distant
Janmaimool and Khajohnmanee, 2020	Sense of obligation (three items; adapted from Gärling et al., 2003)	Moral obligation	Theory/Model: VBN Explanation: Moral obligation is affected by values and influences our behavior. Global citizenship is argued to enhance one’ understanding of interdependence between human and environment, which may lead to formation of altruistic or biospheric views, which are important in the creation of their moral obligation and decision to engage in pro-environmental behavior.
Joanes, 2019	Personal norm (five items*) Awareness of needs (six items*) Ascription of responsibility (six items*) Outcome efficacy (six items*)	Moral obligation responsibility awareness of needs outcome efficacy	Model: NAM + SIT Explanation: global identity is related to prosocial and cooperative behavior based on SIT. It is also found to be associated with interest in events that affect humanity and a felt personal responsibility to better the world, which are linked to the awareness of need and ascription of responsibility in NAM.
Kim et al., 2021	Personal Norm (three items; Scale based on Han et al., 2020) [based on Norm Activation Model (Schwartz, 1977)]. Ascribed Responsibility (three items; scale based on Han, 2014). Psychological Distance (12 items total; 3 items per dimension; Wang et al., 2019)	Moral obligation responsibility relevance	Model: NAM Explanation: Global identity has implications on acting to protect the earth and its people. Climate change means threats to the survival of people within one’s community on Earth. And as such, it would mean the frequent climate phenomena are linked to climate change that is happening currently, everywhere, to anyone and with certainty (i.e., low psychological distance in all dimensions: temporal, spatial, social and hypothetical).
Loy et al., 2021a	Relevance attribution: **Study 1:** Personal relevance (four items**) Societal relevance (four items**) **Study 2:** Relevance attribution to a news article	Relevance	Model: SIT Explanation: Environmental issues are collective interests, and the global identity is relevant in the appraisal of and response to environmental crisis.
Ng and Basu, 2019 (**Study 2** only)	**Study 2:** Personal responsibility (Ng and Basu, 2019) (**Study 1** and **Study 3** did not empirically unpack the relationship between global identity and environment-related construct)	Responsibility	Model: TMR Explanation: TMR focus on personal responsibility and global identity focuses on interconnectedness. Since climate change affect all humans, global identity would lead individuals to feel personally responsible toward the environment.

EMSICA, encapsulated model of social identity in collective action; NAM, norm activation model; SIMCA, social identification model of collective action; SIT, social identity theory; TMR, triangle model of responsibility; TPB, theory of planned behavior; VBN, value-belief-norm model. *All measures were adapted from De Groot and Steg (2009) and Nayum et al. (2016). **Both measures were adapted from Spence and Pidgeon (2010) and Weber and Wirth (2013).

*Obligation* was found in four studies with different labels: “sense of obligation” (Janmaimool and Khajohnmanee, 2020), “personal norm” (Joanes, 2019; Kim et al., 2021) and “moral norm” (Chan et al., 2020). Although obligation took on different names, all four studies referred to it as one’s feeling or sense of obligation to perform a behavior. It was reasoned that individuals with a strong social identity would align their actions with the interests of the group. In the context of environmental problems, individuals with a strong global identity would be more likely to care about the welfare of all humans and be concerned about such problems. Accordingly, strong global identity was conceptualized to be related to a sense of obligation to protect the environment, and in turn, one would display environmental engagement. Some studies have included other motivational factors in predicting PEB engagement, but all studies have conceptualized obligation as the proximal driver behind PEB (Janmaimool and Khajohnmanee, 2020) or PEB intention (Joanes, 2019; Chan et al., 2020; Kim et al., 2021). The findings of these studies support this conceptualization.

*Responsibility* refers to a person’s sense of shared responsibility regarding harmful outcomes. Although both responsibility and obligation refer to a sense of duty, responsibility implies that an individual perceives themselves as part of the cause of a problem and therefore PEB could be seen as reparation of one’s wrongdoing. Meanwhile, obligation implies that an individual is part of the solution to a problem and engaging in PEB contributes to environmental protection. Responsibility was considered in four studies, which either referred to responsibility toward the environment (*n* = 3) or responsibility toward humanity (*n* = 1). The former focused on the negative impacts of human activities on the environment in which all humans live, whereas the latter focused on the negative impacts of human activities on humans directly. It was reasoned that individuals with strong global identity would consider the wellbeing of all humans. Accordingly, strong global identity was theorized to feel a sense of responsibility for the negative impacts of one’s inaction on other humans directly or on the environment in which we live, which, in turn is associated with PEB and/or EC. Indeed, the significant findings of all four studies supported responsibility mediating the relationship between global identity and PEB and/or EC.

*Relevance* refers to how individuals appraise and relate themselves to environmental issues. Three studies considered relevance as a mediator, although they conceptualized relevance using different constructs. Loy et al. (2021a) proposed that global identity is indirectly related to climate change mitigation behavior through relevance attribution. Relevance refers to whether one perceives climate change as interesting, important, relevant, and meaningful. Kim et al. (2021) conceptualized relevance in terms of psychological distance of climate change. According to the construal level theory, four dimensions of psychological distance have been identified: social distance (whether climate change will happen to oneself or others who are socially close or distant), geographical distance (whether climate change will happen geographically close or far away), temporal distance (whether climate change will happen close or far temporally), and hypothetical distance (whether climate change is likely to occur). Regardless of how relevance was defined, climate change was perceived as a threat or crisis. It was theorized that individuals with a strong global identity would take climate change more seriously (Loy et al., 2021a) or be more concerned about the threat of climate change to human survival (Kim et al., 2021). As such, they are more attuned to climate change issues and act to mitigate the threat. The findings of the study by Loy et al. (2021a) support the conceptualization that relevance mediates the relationship between global identity and PEB. Kim et al. (2021) revealed that global identity had a significant direct negative effect on psychological distance and an indirect positive effect on ascribed responsibility *via* psychological distance. However, further analysis revealed that the indirect relationship between global identity and PEB intention was not significant.

It is worth noting that Kim et al. (2021) was the only study that included all three major mediators: personal norm (obligation), ascribed responsibility (responsibility), and psychological distance (relevance). The findings showed that global identity was related to reduced psychological distance of climate change, but it did not indirectly predict PEB *via* any of the three mediators. The structural model results showed that psychological distance had a negative indirect relationship with personal norm *via* ascribed responsibility, and personal norm in turn had a direct effect on environmental behavioral intention. This finding suggests that obligation is most proximal to PEB and responsibility had an indirect relationship with PEB *via* obligation, whereas relevance bridged global identity and responsibility. This observation by Kim et al. (2021) suggest that the mediators may have varying levels of association with global identity and PEB. This contrasts with findings from other studies that considered only one of the three major mediators, where the direct and the indirect relationships of global identity and PEB were significant. While the single-mediator studies are valuable and provide a good start in understanding the specific underlying mechanisms, *we recommend that future studies consider a more complex relationship by exploring the interplay of the major mediators and examine how the findings may compare with those by Kim et al. (2021). [Recommendation #3]*.

Furlong and Vignoles (2021) considered none of the aforesaid major mediators but factors from the social identity model of collective action (van Zomeren et al., 2012) and the encapsulated model of social identity in collective action (Fattori et al., 2015): moral convictions, efficacy, anger, and environmental movement-specific identity. Their final model suggested that global identity predicted participative efficacy, which in turn indirect predicted PEB *via* movement-specific identity. In addition, global identity was related to moral conviction (defined as an absolute stance on a moral issue), and moral conviction predicted anger. This study by Furlong and Vignoles (2021) has two implications for future studies. First, the effects of global identity and relevant mediators on PEB may depend on the type of PEB in question. The PEB in Furlong and Vignoles (2021) was related to a specific environmental movement that can be categorized as environmental activism (Stern, 2000), such as blocking a road during a demonstration and participating in a hunger strike. Echoing the discussion earlier, this is drastically different from operationalizing PEB as daily behavior in the private sphere, be it refraining from flying (Loy et al., 2021b), reducing food waste (Pong, 2021), or aggregating a list of daily PEB, such as recycling and taking shorter showers (e.g., Der-Karabetian et al., 2014). Referring to existing behavioral models, the major motivational factors in collective action (e.g., collective efficacy, negative group-based emotions, moral conviction and social identity in the social identity model of collective action; van Zomeren et al., 2012) are different from the motivational factors underlying private-sphere behavior (e.g., attitude, subjective norm and perceived behavioral control in the theory of planned behavior; Ajzen, 1991). It is possible that the effect of global identity and mediators may vary depending on the pro-environmental outcomes being measured. *We recommend that future studies consider comparing the effect of global identity and the type of mediators based on the type of PEB [Recommendation #4].*

The second implication of Furlong and Vignoles (2021) is related to emotions; it was the only study in this review that considered the role of emotions. The study was based on two models: the social identity model of collective action (van Zomeren et al., 2008) and the encapsulated model of social identity in collective action (Thomas et al., 2012). Both models considered affective injustice, in particular, anger as a group-based emotion in response to injustice. We observe that group-based emotions is a relevant and underexplored area in global identity research. In social identity research, group-based emotions refer to people’s emotional responses to ingroup concerns, as opposed to personal concerns, when their group membership is salient (Yzerbyt et al., 2016). In the context of environmental problems, individuals also experience group-based emotions where they respond emotionally to the responsibility for environmental harm and protection done by their ingroup members, especially when they strongly identify with the group in question (van Zomeren et al., 2008; Harth et al., 2013). Harth et al. (2013) empirically demonstrated that group-based emotions such as anger, pride, and guilt that were directed toward ingroup members were relevant in eliciting different PEBs. For example, anger predicted intentions to punish in-group wrongdoers who have harmed the environment and guilt predicted reparative behavioral intentions such as contributing to repairing the damage to the environment caused by in-group members. These emotions were found to mediate the relationship between in-group responsibility and behavioral intentions.

Another notable feature of Furlong and Vignoles (2021) is that it referred to not only negative emotions (guilt/shame, anger, fear) but also a positive emotion (hope). Although Furlong and Vignoles (2021) did not find hope to be a significant driver of environmental activism, positive emotions may still be relevant to other forms of PEB. Indeed, positive emotions have been linked to PEB in previous studies. For example, pride predicted environmental protection intentions that favored the in-group (Harth et al., 2013). Pride shifts individuals to focus on environmental achievements. Taking responsibility for environmental protection as a group and contemplating the group’s past environmental achievements could lead to the emotion of pride, which, in turn, motivates intention for further action. This previous finding suggests that both negative and positive emotions may have a role to play underlying the behavioral effects of global identity and PEB. *We recommend that researchers explore the role of emotions, including group-based emotions and positive emotions, in the relationship of global identity and PEB [Recommendation #5].*

### 3.4. Additional observations

We made additional observations regarding the research methods used in the reviewed previous research. We now discuss these observations and provide recommendations for future research.

#### 3.4.1. Measurement of global identity

Among the 30 articles reviewed, 17 distinct^1^ global identity measures were identified and nine of them were not covered in previous reviews (McFarland et al., 2019; Reysen, 2022) or empirical analysis (McFarland and Hornsby, 2015). One of the nine measures was an established measure: the “Local-Global Identity” measure by Tu et al. (2012) used in two studies (Ng and Basu, 2019; Kim et al., 2021). The remaining eight measures were created solely for use in specific studies (Iwata, 1996; Russell and Russell, 2010; Strizhakova and Coulter, 2013; Barth et al., 2015; Janmaimool and Khajohnmanee, 2020; Leithead and Humble, 2020; Furlong and Vignoles, 2021; Aydin et al., 2022) and were not used in other studies on this topic.

Different versions of the same measures were observed in addition to the number of distinct measures. For example, the original Identification With All Humanity scale consisted of nine items and was designed with one dimension. A few studies adopted the conceptualization of Identification With All Humanity with two dimensions by Reese et al. (2015) consisting of eight items. While Identification With All Humanity was designed to capture an individual’s stable characteristics (McFarland et al., 2012), one study measured “situational global identity,” that is, how participants thought and felt about global identity after having read a news piece on climate change (Loy et al., 2021a). This modified version of the Identification With All Humanity scale included five items assessing the self-definition dimension (merely identifying oneself as a member) and five items assessing the self-investment dimension (a sense of solidarity) to measure situational global identity. Another study also examined the role of momentary increased salience of global identity, but instead of using a scale to measure momentary salience, they adopted a manipulation by asking participants in the experimental group to unscramble sentences related to global identity (Ng and Basu, 2019).

Unlike all other definitions that considered only humans, two studies referred to a broader conceptualization of the world (Renger and Reese, 2017; Kim et al., 2021). The two studies extended global identity beyond all humans to the Earth, for humans were from nature, and humans were emotionally connected to both human society and the Earth. However, only one measure reflected this definition (Renger and Reese, 2017). The other measure did not explicitly reflect the connection to Earth (Kim et al., 2021).

Although the main objective of this systematic review was to understand the relationship between global identity and the constructs of PEB and EC, it also added to the two past reviews and an analysis of global identity measures in psychology (McFarland and Hornsby, 2015; McFarland et al., 2019; Reysen, 2022). Together, the three articles covered 10 unique measures, and the systematic search in the present review yielded seven additional distinct global identity measures. Several of these measures were published after 2010. It is exciting to witness the burgeoning development and use of global identity measures in the past decade. That said, there seem to have been overlapping research efforts in this area. This is an unavoidable problem for an area of research that is still in its infancy. *We recommend that future research empirically examines the convergence and divergence of this wide variety of measures and thereby identify the best way of assessing global identity [Recommendation #6].*

#### 3.4.2. Measurement of PEB and EC

A variety of environment-related constructs were used to operationalize the outcomes. We coded each measure according to type. For example, a scale that measures intention to reduce clothing consumption (Joanes, 2019) would be coded as “intentions.” Alternatively, if participants were asked if they were willing to pay for materials printed on recycled paper (Ng and Basu, 2019) would be coded as “willingness to pay.” Twenty types of measures were found. Some studies included more than one type, and 50 measures were used in the 35 studies.

The most common types were behavior-related measures, including behavioral intentions (*n* = 11 studies), self-report on-going habits (*n* = 11), policy support (*n* = 4), willingness to pay (*n* = 4), self-report past behavior (*n* = 3), behavior proxy (*n* = 3), and environmental activism (*n* = 1). Together, these accounted for over 60% of the total measures used in the studies.

Most studies adopted self-report measures. Only one study used observational measures in a laboratory setting as indicators of behavioral intentions (Loy et al., 2021a). Two measures were employed during the study: information viewing time and budget allocation.

Due to the intention-behavior gap (Bamberg and Möser, 2007) and social desirability effect (Vesely and Klöckner, 2020), it is important to examine the effect of global identity on actual behavior with real environmental and personal consequences, and to understand the underlying mechanisms to mobilize global efforts to tackle environmental problems and climate change. For example, researchers may consider observations of behavior in the field using either observers or devices such as smart meters that measure energy consumption (Lange and Dewitte, 2019). Researchers may also consider employing the pro-environmental behavior task developed by Lange et al. (2018) to observe behavior with actual personal time costs in a laboratory setting. *We recommend future studies go beyond measuring behavioral intentions and self-report behavior and consider measuring observations in the laboratory or in the field [Recommendation #7].*

#### 3.4.3. Sampling

**FIGURE 2:**
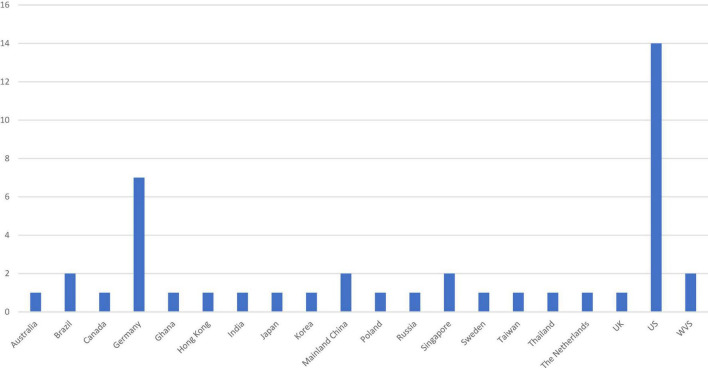
Region/country in which data was collected. WVS stands for World Value Survey (2005–2009). Two studies used data from WVS [**Study 1** in Ng and Basu (2019); Running, 2013].

We examined the demographics of the samples by coding the country of origin and age group of each study. We chose to identify the country in which the data collection occurred rather than to code the nationality of the participants because of an overall lack of information on the composition of the participants in terms of ethnicity and nationality. Excluding three studies that used data from the World Value Survey (Wave 5, 2005–2009 and/or Wave 6, 2010–2014) in which samples from dozens of countries were drawn, we found that the remaining 32 studies drew samples from a total of 19 different countries (some from multiple countries in the same study), with the US in the lead (15 samples), followed by Germany (7 samples), and all other countries represented only once or twice (Figure 2).

The dominance of Western, educated, industrialized, rich, and democratic (WEIRD) countries (Henrich et al., 2010) in the drawn samples was apparent in other ways: Even when some studies recruited participants from multiple countries, they recruited from Western countries only and countries and regions from other parts of the world were underrepresented. Although the education level distribution of the community samples was largely unavailable, close to half of the studies (14 of the 32 studies) consisted solely of university students as participants. They reported a mean age of 19.36 to 32 years, with two studies reporting a mean age under 20 years, one over 30 years, and the majority of them under 25. Almost two-thirds of these countries are highly developed with a high literacy rate and national income, as indicated by the Human Development Index scores of 0.80 and above (United Nations Development Programme, 2020). Some studies recruited community samples (11 studies) or used a mixed sample of students and community members (5 studies). Nonetheless, the mean age of the participants hovered around 30 to 40 years of age in the community samples. Thus, different generations can be better represented.

This finding of dominance of WEIRD countries is in line with past observations (Arnett, 2002; Tam and Milfont, 2020; Tam et al., 2021). Although this issue is already widely known in psychological and behavioral research, we believe it is of particular importance to this topic, not only because environmental problems concern every population in the world, but also because there is some preliminary evidence on the potential cross-national variability of the relationship between global identity and PEB and EC (Der-Karabetian et al., 2014; Der-Karabetian and Alfaro, 2015). *We recommend that future studies expand the geographic and demographic representation of their samples; accurate and detailed reporting of sample characteristics is also encouraged [Recommendation #8].*

## 4. General discussion

With growing research interest in the relationship between global identity and environmentalism, there is a need to integrate and synthesize existing research. The present systematic review aimed to meet this need by reviewing the empirical evidence regarding this relationship and examining its theoretical underpinnings. In the following section, we elaborate on the theoretical significance of the synthesized evidence. We will also summarize our recommendations made previously in correspondence with our key observations and propose two additional ones (concerning the potential dynamics between global identity and other forms of social identity, and emotional burden, specifically in the form of climate anxiety, as a potential psychological consequence of global identity).

### 4.1. Theoretical significance

Findings in this systematic review reveal strong support to the notion that individuals with stronger global identity have greater EC and are more likely to engage in PEB, which benefit not only the wellbeing of the planet but also the welfare of all humans. These findings are in line with what the social identity theory and self-categorization theory suggest: When identifying with a group, individuals become concerned about the welfare of fellow group members and are more likely to act in the interest of the group. Findings from a small subset of the reviewed studies further support this notion by uncovering the psychological mechanisms underlying the link between global identity and PEB and EC. They referred to such relevant constructs as obligation, responsibility, and relevance, and revealed viable ways to integrate the social identity perspective with other psychological theories and models (e.g., the theory of planned behavior, psychological distance, the norm activation model, the social identity model of collective action).

It should be noted that the role of social identity has been widely discussed in research on humans’ responses to environmental issues. Social identity plays as a centerpiece in such models as the social identity model of collective action (van Zomeren et al., 2008; Bamberg et al., 2015), the encapsulated model of social identity in collective action (Fattori et al., 2015) and, most recently, the social identity model of pro-environmental action (Fritsche et al., 2018). These models similarly suggest that social identity is a key driver behind engagement in collective environmental actions. The findings reviewed in the current systematic review expand these models in the following ways. First, they not only affirm that social identity is a key driver behind environmental engagement but also highlight that the types of social identity that bear environmental implications are not limited to only groups that are explicitly associated with the environmental movement (e.g., environmentalists, a specific environmental campaign), which have been the typical focus in the aforementioned models. Our findings suggest that global identity, a highly inclusive type of identity that does not bear any apparent association with environmentalism, could also bear a significant effect on pro-environmental outcomes. Second, the studies included in the present review considered various types of pro-environmental outcomes that are not limited to only collective action, the main focus in some of the aforementioned models. Global identity has been shown to be associated with a wide range of environmental behavior, including not only collective environmental action such as environmental protests but also private-sphere behavior and policy support.

Third, while the aforementioned models commonly consider group-based grievance and hence anger as a potential mechanism behind the effect of social identity on engagement in collective environmental action, the present review suggests the potential roles of other emotions. For example, in a few studies, one’s sense of responsibility for causing cause environmental problems was found to be a mediator behind the effect of global identity and PEB and EC. It is conceivable to expect that guilt, which is often associated with the recognition of one’s own wrongdoings and the motivation to repair damages (Harth et al., 2013), could be another emotion that underlies the effect of global identity on PEB and EC. Positive emotions such as hope (Kleres and Wettergren, 2017) and pride (Harth et al., 2013) could be relevant too. It is argued that individuals with stronger global identity may feel a stronger sense of hope and optimism and experience more pride as they reflect on the progress and achievements humans have made so far in the mitigation against environmental problems (e.g., the Paris Agreement, advances in environmental technology).

Taken together, the current review reveals some new nuances regarding how social identity is associated with pro-environmental outcomes. In the following, building upon these new nuances, we recommend some important directions for future research on the topic.

### 4.2. Future research directions

#### 4.2.1. Recommendations already made

In the discussion in the previous section, we identified eight recommendations for future research. The results are summarized in Table 3. Overall, these recommendations can be categorized into three groups: understanding the relationship between global identity and PEB and EC; expanding the main constructs; and sampling considerations.

**TABLE 3:** Summary of recommendations.

Recommendation #	Section origin	Description of recommendation	Reason(s)
#1	3.1. Evidence on the positive relationship between global identity and PEB and EC	Consider diverse sampling to explore potential national/cultural differences in the relationship	Cross-national differences found in the relationship between global identity and PEB and EC (some relationships were non-significant; Der-Karabetian et al., 2014; Der-Karabetian and Alfaro, 2015)
#2	3.1. Evidence on the positive relationship between global identity and PEB and EC	Explore the indirect effect of global identity by considering the underlying mechanisms of the relationship between global identity and PEB/EC	A non-significant direct relationship of global identity and PEB but a significant indirect relationship (Chan et al., 2020; Furlong and Vignoles, 2021)
#3	3.3. Evidence on the mediating mechanisms underlying the positive relationship	Explore the interplay between the three major mediators (obligation, responsibility and relevance)	Findings showed that mediators had varying levels of association with global identity and PEB (Joanes, 2019; Kim et al., 2021)
#4	3.3. Evidence on the mediating mechanisms underlying the positive relationship	Compare the effect of global identity and the type of mediators based on the type of PEB	Different theoretical models with different mediators were used to explain different types of PEB, such as collective action model with anger and moral conviction was used in environmental activism (Furlong and Vignoles, 2021), and theory of planned behavior with attitude and obligation in private PEB (Chan et al., 2020).
#5	3.3. Evidence on the mediating mechanisms underlying the positive relationship	Explore the role of emotions, group-based emotions, moral emotions, and positive emotions, in the relationship of global identity and PEB	Findings showed that anger played a role in mediating global identity and environmental activism (Furlong and Vignoles, 2021). This is the only study that considered emotions as a mediator. Emotions are underexplored in the study of the relationship between global identity and PEB and EC
#6	3.4.1. Measurement of global identity	Examine how the various global identity measures converge and diverge	In the 32 articles reviewed, 17 distinct global identity measures were identified. Different versions of the same scales were found (Identification With All Humanity, McFarland and Hornsby, 2015; Global Citizenship Identification, Reysen and Katzarska-Miller, 2013)
#7	3.4.2. Measurement of PEB and EC	Go beyond measuring behavioral intentions and self-report behavior and consider measuring observations in the laboratory or in the field	Over 60% of the studies adopted behavior-related measures and all studies employed surveys to measure PEB and EC with one exception: Only one study (Loy et al., 2021a) used observations in the laboratory
#8	3.4.3. Sampling	Expand the geographic and demographic representation of samples; accurate and detailed reporting of sample characteristics is also encouraged	Dominance of WEIRD countries in sampling. Mean ages of participants were 30 to late 40 s.
#9	4.2.2. Two additional recommendations	Pay close attention to the dynamics between global identity and other social identities and test their effects on PEB and EC in the context of specific environmental problems and issues wherein these different types of social identity may generate differential effects	A person tends to have multiple group memberships and the interests of these groups may conflict with each other. This conflict of interests may have implications on PEB and EC.
#10	4.2.2. Two additional recommendations	Explore maladaptive responses as a potential outcome of global identity	Climate anxiety is a psychological response to climate change (Clayton and Karazsia, 2020). Since strong global identity were related to obligation, responsibility and relevance, it would be important to explore the psychological burden of global identity

**FIGURE 3:**
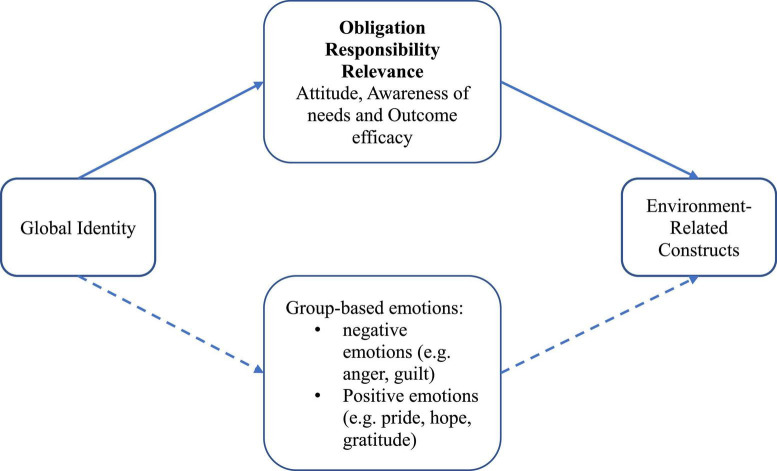
Tested and proposed underlying mechanisms. The box with solid lines at the top of the diagram houses the underlying mechanisms tested in the nine studies reviewed. The three mechanisms in bold are the three major themes. The box with the dash lines at the bottom of the diagram showed proposed underlying mechanisms.

The findings of this review suggest that the relationship, though robustly positive, is not always straightforward. This relationship is not necessarily consistent across societal contexts (e.g., Der-Karabetian et al., 2014; Der-Karabetian and Alfaro, 2015). An indirect effect of global identity could exist, even in the absence of a significant direct effect (e.g., Chan et al., 2020; Furlong and Vignoles, 2021). Multiple mediators underlying this relationship are likely to be involved (e.g., Joanes, 2019; Kim et al., 2021). In addition, the strength of the relationship and type of mediating factors may vary depending on the type of PEB (e.g., Chan et al., 2020; Furlong and Vignoles, 2021). Emotions could play a significant role in this relationship; however, they have been understudied. Accordingly, we recommend that future studies explore between-society variability in the relationship (*Recommendation #1*), explore the potential underlying mediating mechanisms (*Recommendation #2*), consider the interplay of multiple mediators (*Recommendation #3*), examine the extent to which the relationship depends on the type of PEB (*Recommendation #4*), and explore the role of emotions, including group-based emotions and moral emotions, both positive and negative, in the relationship (*Recommendation #5*). We have summarized the tested mediators of the relationship and the proposed mediators of emotions in Figure 3.

The second set of recommendations concerns the measurement of the two main constructs. For global identity, we observed heterogeneity in the measures for the construct, where a total of 17 distinct measures were identified. In contrast, PEB and EC were almost exclusively assessed using self-report measures, and over 60% of the measures were behavior related. We recommend that future studies examine how the various measures of global identity converge and diverge (*Recommendation #6*) and use observational measures of actual behavior (*Recommendation #7*).

The final recommendation concerns sampling. As mentioned, there appear to be cross-national differences in the relationship between global identity and PEB and EC. We also observed that participants from WEIRD countries dominated the samples in the reviewed studies; participants tended to be in their 20 s for university samples and in their 30 s to late 40 s for community samples. We recommend that future studies diversify the geographic and demographic representation of their samples (*Recommendation #8*).

#### 4.2.2. Two additional recommendations

In addition to the aforementioned recommendations, which correspond to the key observations reported earlier, we make the following two recommendations:

People tend to have multiple group memberships (Turner et al., 1987; McFarland et al., 2012). It is possible that the interests of multiple groups a person identifies with at the same time conflict with each other. One may identify with all humanity (global identity) as well as one’s neighborhood (community identity), and the interests of humanity as a whole may conflict with those of the local neighborhood. The Not-In-My-Backyard phenomenon is an illustrative example (e.g., Devine-Wright, 2009). Despite popular public support for renewable energy in opinion polls, renewable energy projects are often met with fierce opposition from residents. These projects may be perceived as disruptions to the neighborhood and the local community, and hence, a threat to one’s community identity. In other words, while supporting renewable energy projects would be in line with a person’s support for climate efforts based on their global identity, it may contradict their local community identity. Aydin et al. (2022) empirically examined this line of thought. They compared nationalism (a negative form of national identification related to right-wing authoritarianism and social dominance orientation), patriotism (a positive form of national identification related to place attachment), and global identity. They found that patriotism and global identity were positively related to pro-environmental beliefs and PEB, whereas nationalism was negatively related to pro-environmental beliefs and activism. This observation implies that the effect of global identity on environmentalism should be situated within the context of the effects of other types of social identity. *We recommend that future studies pay close attention to the dynamics between global identity and other social identities and test their effects on PEB and EC in the context of specific environmental problems and issues wherein these different types of social identity may generate differential effects [Recommendation #9].*

As seen in our review, the existing research on the environmental implications of global identity has tended to focus solely on its positive effects; that is, previous studies have mostly focused on the extent to which global identity motivates people to respond actively and adaptively to various environmental problems, such as climate change, in the form of PEB. However, it should be noted that humans could also respond to environmental problems in a maladaptive manner. For instance, emerging research shows that climate change can have a negative impact on mental health even among people who do not have a direct experience of climate-related extreme events or disasters *via* the effect of climate change anxiety (Clayton and Karazsia, 2020) or eco-anxiety (Stanley et al., 2021). Findings show that higher climate change anxiety is associated with poorer mental health, including more clinically significant anxiety and depressive symptoms, lower levels of psychological wellbeing, and higher levels of psychological distress and ill-being (e.g., Hickman et al., 2021; Schwartz et al., 2022). In response to the experienced and anticipated impacts of climate change, some people develop cognitive and emotional impairments (e.g., difficulty in concentrating and having nightmares) and functional impairment (e.g., difficulty at work and in interpersonal relationships; Clayton and Karazsia, 2020). Given that global identity represents a greater concern for the environment and the wellbeing of fellow humans, it is conceivable that it can lead to a greater psychological burden and emotional toll in the form of anxiety, impairment, and other mental symptoms as people face climate change and many other environmental problems. To the best of our knowledge, this possibility has not yet been explored in the literature. *We recommend that future research considers maladaptive responses as a potential outcome of global identity [Recommendation #10].*

## 5. Conclusion

The interconnectedness and common fate among humans, as well as the interdependence between humans and the environment, suggest the imperative to consider an all-inclusive, superordinate identity that can unite everyone in the world together in the battle against global problems such as climate change and environmental crises. The present systematic review exposes the empirical validity of this contention and highlights gaps that are yet to be filled by future research. We call upon researchers across disciplines to consider our recommendations and continue to build a nuanced understanding of this topic.

## Data availability statement

The original contributions presented in this study are included in the article/Supplementary material, further inquiries can be directed to the corresponding author.

## Author contributions

VP: data curation, formal analysis, methodology, project administration, validation, visualization, writing—original draft preparation, and writing—review and editing. K-PT: conceptualization, methodology, project administration, and writing—review and editing. Both authors contributed to the article and approved the submitted version.

## Footnotes

1 By distinct we mean any adapted version was not counted as a separate scale. For example, different versions of the Identification With All Humanity scale that had been used, from 8 items to 10 items in different languages, were counted as one distinct measure. Similarly, the Global Citizenship Identification by Reysen and Katzarska-Miller (2013) had versions of 2 items, 22 items, and one combined with items from another scale; all adapted versions were counted as one distinct measure.
