# Supplementary material for: Relationship between global identity and pro-environmental behavior and environmental concern: a systematic review
Source: Front Psychol. 2023 Apr 17;14:1033564. doi: 10.3389/fpsyg.2023.1033564 (PMC10149791; doi:10.3389/fpsyg.2023.1033564)
Supplement: Supplementary file 1 [file Data_Sheet_1.docx]

**Understanding the Relationship between Global Identity and Pro-Environmental Behavior and Concern: A Systematic Review**

Vivien Pong, & Kim-Pong Tam

**Supplementary Materials**

**S1. Search Terms**

Global Identity Search Terms

“all humans everywhere” OR “people all over the world” OR “people from different countries around the world” OR “global citizen*” OR “citizen of the world” OR “citizens of the world” OR “members of world community” OR “all humanity” OR “Psychological Sense of Global Community” OR “Sense of World Community” OR “World citizenship” OR “Global Social Identity” OR “Global Citizenship Identification” OR “World citizen” OR “Global Social Identity” OR “Global Social Identification” OR “Global Belonging” OR “Local-Global Identity” OR “Human Esteem” OR “Global Identity” OR “Human Identity” OR “Common Human Identity” OR “members of the world” OR “members of the global community” OR “members of the global*” OR “global community” OR “world citizen*” OR “Identification With All Humanity”

Environment-Related Construct Search Terms

“green consumer behav*” OR “green behav*” OR “sustainable consumer behav*” OR “sustainable behav*” OR “environmental consumer behav*” OR “environmental behav*” OR “ecological consumer behav*” OR “ecological behav*” OR “energy behav*” OR “pro-environmental consumer behav*” OR “pro-environmental behav*” OR “proenvironmental consumer behav*” OR “proenvironmental behav*” OR “proenvironment*” OR “environmentally friendly behav*” OR “environmentally friendly” OR “environmentally conscious behav*” OR “environmentally related behav*” OR “environmentally related” OR “public transport behav*” OR “waste recycling behav*” OR “waste recycling” OR “recycling behav*” OR “recycl*” OR “environmentally significant behav*” OR “conservation” OR “pro-environmental action” OR “environmentally responsible behav*” OR “ecologically responsible behav*” OR “environmentally friendly behav*” OR “sustainable action” OR “environmental action” OR “pro-environmental collective action” OR “environmental activism” OR “support for environmental policy” OR “environmental policy support” OR “environmental stewardship” OR “conservation behav*” OR “environmental engagement” OR “waste reduction” OR “energy saving” OR “energy conservation” OR “travel mode choice” OR “pro-environmental mobility behaviour” OR “water conservation” OR “green consumerism” OR “green purchases” OR “ecological consumer behaviour” OR “green consumer behaviour” OR “environment protection behaviour” OR “Proenvironmental intentions” OR “environmentally conscious” OR “environmentally responsible” OR “reuse” OR “green” OR “renewable” OR “PEB” OR “GEB” OR “climate change” OR “global warming” OR “environmental attitude*” OR “pro-environmental attitude*” OR “environmental concern” OR “Environmental performance” OR “Environmental sustainability” OR “Environmental concern” OR “Green behav*” OR “Pro-environmental behav*” OR “environmental value*” OR “ecologic value*”

**S2. Search Term String Length Test**

As there might be limitations to the search string length in the selected databases (Gusenbauer & Haddaway, 2020), we did the following to test the limitations of each named database: We first added a unique identifiable search term in quotation as the last search term in the string of each set of search terms. If articles related to this unique search term appeared, it meant that the search string length for this search did not hit the upper limit in the database. On the other hand, if no article returned on this unique search term, five terms were deleted at a time until there were returns. Then, a term was added at a time to find the length limit.

The results of this search term string length test were that, for all **EBSCOhost databases** (Academic Search Premier, APA PsycINFO, APA PsycARTICLES, ERIC, GreenFILE), ProQuest and Web of Science, the current search term string length did not find the string length limit (search term length = 71 terms). For **Scopus**, if the “Advanced document search” option was used, the current search term string length did not find the Scopus limit. However, if the search bars on the homepage were used, the string length limit was found to be 47 search terms.

**References**

Gusenbauer, M., & Haddaway, N. R. (2020). Which academic search systems are suitable for systematic reviews or meta‐analyses? Evaluating retrieval qualities of Google Scholar, PubMed, and 26 other resources. Research synthesis methods, 11(2), 181-217.

**S3. Search Settings and Related Details**

Search on Apr 13, 2022:

Database: EBSCOhost (APA PsycINFO and APA PsycARTICLES)

Global Identity search terms [Abstract] AND PEB search terms [Abstract]

Expanders: Apply equivalent subjects

Limiters: Scholarly (Peer Reviewed) Journal, Exclude Book Reviews, English, Journal Article, Exclude Dissertation, Human/Male/Female (exclude animal)

Returned Results: 34 (duplicates removed by search engine)

Database: EBSCOhost (APA PsycINFO and APA PsycARTICLES)

Global Identity search terms [Title] AND PEB search terms [Title]

Expanders: Apply equivalent subjects

Limiters: Scholarly (Peer Reviewed) Journal, Exclude Book Reviews, English, Journal Article, Exclude Dissertation, Human/Male/Female (exclude animal)

Returned Results: 10

Database: EBSCOhost (APA PsycINFO and APA PsycARTICLES)

Global Identity search terms [Subject Terms] AND PEB search terms [Subject Terms]

Expanders: Apply equivalent subjects

Limiters: Scholarly (Peer Reviewed) Journal, Exclude Book Reviews, English, Journal Article, Exclude Dissertation, Human/Male/Female (exclude animal)

Returned Results: 13

Database: EBSCOhost (Academic Search Premier, ERIC, GreenFILE)

Global Identity search terms [ABS] AND PEB search terms [ABS]

Expanders: Apply equivalent subjects

Limiters: Scholarly (Peer Reviewed) Journal, Article, Periodical, Journal Article, English, Academic Journal

Returned Results: 134 (duplicates removed by search engine)

Database: EBSCOhost (Academic Search Premier, ERIC, GreenFILE)

Global Identity search terms [TI] AND PEB search terms [TI]

Expanders: Apply equivalent subjects

Limiters: Scholarly (Peer Reviewed) Journal, Article, Periodical, Journal Article, English, Academic Journal

Returned Results: 17 (duplicates removed by search engine)

Database: EBSCOhost (Academic Search Premier, ERIC, GreenFILE)

Global Identity search terms [SU] AND PEB search terms [SU]

Expanders: Apply equivalent subjects

Limiters: Scholarly (Peer Reviewed) Journal, Article, Periodical, Journal Article, English, Academic Journal

Returned Results: 13 (duplicates removed)

Database: Scopus

Global Identity search terms [Abstract] AND PEB search terms [Abstract]

Limiters: Journal, Article, English, Subject Area

Returned Results: 280

Database: Scopus

Global Identity search terms [Title] AND PEB search terms [Title]

Limiters: Journal, Article, English, Subject Area

Returned Results: 20

Database: Scopus

Global Identity search terms [Keywords] AND PEB search terms [Keywords]

Limiters: Journal, Article, English, Subject Area

Returned Results: 35

Database: ProQuest

Global Identity search terms [Abstract] AND PEB search terms [Abstract]

Limiters: Peer Reviewed, Scholarly Journals, Reports, Article, Report, Review, English, “shows results outside of my library subscription”

Returned Results: 281

Database: ProQuest

Global Identity search terms [Title] AND PEB search terms [Title]

Limiters: Peer Reviewed, Scholarly Journals, Reports, Article, Report, Review, English, “shows results outside of my library subscription”

Returned Results: 12

Database: Web of Science

Global Identity search terms [Abstract] AND PEB search terms [Abstract]

Limiters: Articles, Reviewed Articles, Exclude Proceedings Papers, English

Returned Results: 204

Database: Web of Science

Global Identity search terms [Title] AND PEB search terms [Title]

Limiters: Articles, Reviewed Articles, Exclude Proceedings Papers, English

Returned Results: 15

Database: Web of Science

Global Identity search terms [Keywords] AND PEB search terms [Keywords]

Limiters: Articles, Reviewed Articles, Exclude Proceedings Papers, English

Returned Results: 1

Total articles found = 1069; Duplicates = 462

**S4. Additional Search with New Search Terms**

**Global Identity: Search Terms Discovered during Review Process:**

“all humans everywhere” OR “people all over the world”

**PEB and Environmental Concern Search Terms from April 13, 2022:**

“green consumer behav*” OR “green behav*” OR “sustainable consumer behav*” OR “sustainable behav*” OR “environmental consumer behav*” OR “environmental behav*” OR “ecological consumer behav*” OR “ecological behav*” OR “energy behav*” OR “pro-environmental consumer behav*” OR “pro-environmental behav*” OR “proenvironmental consumer behav*” OR “proenvironmental behav*” OR “proenvironment*” OR “environmentally friendly behav*” OR “environmentally friendly” OR “environmentally conscious behav*” OR “environmentally related behav*” OR “environmentally related” OR “public transport behav*” OR “waste recycling behav*” OR “waste recycling” OR “recycling behav*” OR “recycl*” OR “environmentally significant behav*” OR “conservation” OR “pro-environmental action” OR “environmentally responsible behav*” OR “ecologically responsible behav*” OR “environmentally friendly behav*” OR “sustainable action” OR “environmental action” OR “pro-environmental collective action” OR “environmental activism” OR “support for environmental policy” OR “environmental policy support” OR “environmental stewardship” OR “conservation behav*” OR “environmental engagement” OR “waste reduction” OR “energy saving” OR “energy conservation” OR “travel mode choice” OR “pro-environmental mobility behaviour” OR “water conservation” OR “green consumerism” OR “green purchases” OR “ecological consumer behaviour” OR “green consumer behaviour” OR “environment protection behaviour” OR “Proenvironmental intentions” OR “environmentally conscious” OR “environmentally responsible” OR “reuse” OR “green” OR “renewable” OR “PEB” OR “GEB” OR “climate change” OR “global warming” OR “environmental attitude*” OR “pro-environmental attitude*” OR “environmental concern” OR “Environmental performance” OR “Environmental sustainability” OR “Environmental concern” OR “Green behav*” OR “Pro-environmental behav*” OR “environmental value*” OR “ecologic value*”

**Search Settings and Related Details regarding the Additional Search:**

*All settings (i.e. expanders and limiters) and databases used were the same as the main search completed on April 13, 2022, except that the publication month and year were set to be in or before April, 2022. In the databases where the month could not be specified, the articles were screened manually.

| **Database** | **Search terms [Search Field]** | **Returned Results** |
| --- | --- | --- |
| EBSCOhost (APA PsycINFO and APA PsycARTICLES) | “all humans everywhere” OR “people all over the world” [Abstract] AND PEB search terms [Abstract] | 1 |
|  | “all humans everywhere” OR “people all over the world” [Title] AND PEB search terms [Title] | 0 |
|  | “all humans everywhere” OR “people all over the world” [Subject Terms] AND PEB search terms [Subject Terms] | 0 |
| EBSCOhost (Academic Search Premier, ERIC, GreenFILE) | “all humans everywhere” OR “people all over the world” [ABS] AND PEB search terms [ABS] | 9 |
|  | “all humans everywhere” OR “people all over the world” [TI] AND PEB search terms [TI] | 0 |
|  | “all humans everywhere” OR “people all over the world” [SU] AND PEB search terms [SU] | 0 |
| Scopus | “all humans everywhere” OR “people all over the world” [Abstract] AND PEB search terms [Abstract] | 20 |
|  | “all humans everywhere” OR “people all over the world” [Title] AND PEB search terms [Title] | 0 |
|  | “all humans everywhere” OR “people all over the world” [Keywords] AND PEB search terms [Keywords] | 1 |
| ProQuest | “all humans everywhere” OR “people all over the world” [Abstract] AND PEB search terms [Abstract] | 9 |
|  | “all humans everywhere” OR “people all over the world” [Title] AND PEB search terms [Title] | 0 |
| Web of Science | “all humans everywhere” OR “people all over the world” [Abstract] AND PEB search terms [Abstract] | 0 |
|  | “all humans everywhere” OR “people all over the world” [Title] AND PEB search terms [Title] | 0 |
|  | “all humans everywhere” OR “people all over the world” [Keywords] AND PEB search terms [Keywords] | 0 |
|  | Total | 40 |
|  | Excluded due to being published after April, 2022 | 2 |
|  | Total included for Screening (Stage 2 in PRISMA) | 38 |
